# Cardiomyocyte Hypocontractility and Reduced Myofibril Density in End-Stage Pediatric Cardiomyopathy

**DOI:** 10.3389/fphys.2017.01103

**Published:** 2017-12-22

**Authors:** Ilse A. E. Bollen, Marijke van der Meulen, Kyra de Goede, Diederik W. D. Kuster, Michiel Dalinghaus, Jolanda van der Velden

**Affiliations:** ^1^Department of Physiology, Amsterdam Cardiovascular Sciences, VU University Medical Center, Amsterdam, Netherlands; ^2^Department of Pediatric Cardiology, Erasmus Medical Center, Erasmus University Rotterdam, Rotterdam, Netherlands; ^3^Netherlands Heart Institute, Utrecht, Netherlands

**Keywords:** pediatrics, heart failure, titin, myofibril density, hypocontractility, cardiomyopathy, dilated

## Abstract

Dilated cardiomyopathy amongst children (pediatric cardiomyopathy, pediatric CM) is associated with a high morbidity and mortality. Because little is known about the pathophysiology of pediatric CM, treatment is largely based on adult heart failure therapy. The reason for high morbidity and mortality is largely unknown as well as data on cellular pathomechanisms is limited. Here, we assessed cardiomyocyte contractility and protein expression to define cellular pathomechanisms in pediatric CM. Explanted heart tissue of 11 pediatric CM patients and 18 controls was studied. Contractility was measured in single membrane-permeabilized cardiomyocytes and protein expression was assessed with gel electrophoresis and western blot analysis. We observed increased Ca^2+^-sensitivity of myofilaments which was due to hypophosphorylation of cardiac troponin I, a feature commonly observed in adult DCM. We also found a significantly reduced maximal force generating capacity of pediatric CM cardiomyocytes, as well as a reduced passive force development over a range of sarcomere lengths. Myofibril density was reduced in pediatric CM compared to controls. Correction of maximal force and passive force for myofibril density normalized forces in pediatric CM cardiomyocytes to control values. This implies that the hypocontractility was caused by the reduction in myofibril density. Unlike in adult DCM we did not find an increase in compliant titin isoform expression in end-stage pediatric CM. The limited ability of pediatric CM patients to maintain myofibril density might have contributed to their early disease onset and severity.

## Introduction

### Disease progression in pediatric cardiomyopathy

Dilated cardiomyopathy (DCM) is a cardiac disease characterized by dilatation of the (left) ventricle and systolic dysfunction. A genetic cause is found in 20–50% of DCM cases (Hershberger et al., 2010; Herman et al., 2012; van Spaendonck-Zwarts et al., 2013). The disease often develops in adulthood and treatment is currently only able to slow down disease progression (Hershberger et al., 2013). In children, DCM is a rare disease with an annual incidence estimated around 0.6/100,000 (Towbin et al., 2006). In two large studies the 1- and 5-year transplantation-free survival rates have been reported to be only 69–74 and 54–65%, respectively (Towbin et al., 2006; Alexander et al., 2013). The rate of severe adverse events is low 5 years after presentation (Alexander et al., 2013) and most children die or receive cardiac transplantation because of pump failure. The 5-year cumulative incidence rate of sudden cardiac death is relatively low (2.4%; Pahl et al., 2012). Poor prognosis in pediatric DCM patients was associated with thin left ventricular (LV) wall (LV posterior wall thickness z-score < − 1.7) and age <13.1 years at time of diagnosis (Towbin et al., 2006; Pahl et al., 2012; Alexander et al., 2013). In contrast to adult DCM, pediatric CM can be reversible as in 22% of patients a full recovery has been reported (Everitt et al., 2014). The aggressive nature of pediatric DCM and the contradictory relative high recovery rate was also reported in a recent Dutch study with a 1- and 5-year survival rate of 85 and 84%, respectively, which implies that most patients died within the first year after diagnosis (den Boer et al., 2015). A low transplantation rate in the 1st year after presentation and a 38% recovery rate, of which 50% within 1 year, was reported in this study as well (den Boer et al., 2015). These studies together show that there's little difference in mortality after 1 and after 5 years in pediatric CM which implies that most events occur in the first year. This is different compared to adult DCM in which a slow disease progression and low recovery rate is more common (Felker et al., 2000).

### Pathogenesis of pediatric cardiomyopathy

Determinants underlying the highly diverse response to therapy, recovery rates and mortality are largely unknown. While several pathomechanisms have been elucidated in adult DCM, knowledge of pathogenesis which underlies the aggressive progression of DCM at young age is scarce. A recent study showed that pediatric DCM patients show a different gene expression profile compared to adult onset DCM (Tatman et al., 2017). This study showed that pediatric CM samples were characterized by an expression profile that reflected a more undifferentiated cellular state, and showed a reduced hypertrophic response compared to adult DCM (Tatman et al., 2017). In order to understand why pediatric CM patients show diverse responses to therapy and different recovery and mortality rates compared to adult DCM, it is important to understand which cellular processes are changed in pediatric CM. However, since pediatric CM is rare and cardiac tissue from these patients is scarce, limited information is available on the cellular pathogenesis which underlies this disease.

In this study we explored the cellular phenotype of end-stage pediatric CM samples. We combined functional measurements in single isolated cardiomyocytes with protein analyses and electron microscopy in a unique collection of pediatric CM samples. Our studies revealed greatly reduced force generating capacity of single cardiomyocytes caused by a significant reduction in myofibril density. We observed troponin I hypophosphorylation which was associated with increased myofilament Ca^2+^-sensitivity and impaired myofilament length-dependent activation. We did not find an increase in compliant titin isoform in the pediatric samples, which is a common form of disease remodeling in various forms of adult heart failure (Makarenko et al., 2004; Nagueh et al., 2004; Bollen et al., 2017b). However, we did find a large variation in titin isoform composition in the pediatric CM group. Overall, our data indicate that the pediatric CM heart that progresses to end-stage failure has limited capacity to adequately respond to increased wall stress.

## Methods

### Clinical characteristics

Echocardiographic examinations were performed in a uniform way. All children were at rest and in sinus rhythm during examination and a complete 2-dimensional echocardiographic study was performed. M-mode of the parasternal long-axis was used to measure LV posterior wall in systole (LVPWs), LV posterior wall in diastole (LVPWd), LV end diastolic diameter (LVEDD), and LV end systolic diameter (LVESD) which were expressed as Z-score for body surface area. Subsequently, fractional shortening (FS) was calculated using the formula [(LVEDD − LVESD)/LVEDD] ^*^100%.

### Cardiomyocyte force measurements

Single cardiomyocytes were mechanically isolated from cardiac tissue and membrane-permeabilized as previously described (van Dijk et al., 2012). In short, 10–15 mg of tissue was defrosted in 4°C isolation relax solution containing 1 mM free Mg, 139.6 mM KCl, 2 mM EGTA, 5.95 mM ATP, and 10 mM imidazole with pH adjusted to 7.0 with KOH. The tissue was mechanically disrupted with a Teflon piston for 5–10 s at 900 g to obtain a suspension of single cells, small clumps of cells and cell fragments. The cells were incubated with 0.5% TritonX-100 (Millipore) for 5 min at 4°C to permeabilize the membranes. Triton was removed from cell suspension by 3 subsequent washes with isolation relax solution. Cells were kept at 4°C until measurement on the same day. Single cardiomyocytes were glued with silicon based glue (DB-025, Zwaluw, Den Braven) between stainless steel needles attached to a force transducer (Memscap, AE801) and a length motor with length controller (Aurora Scientific Inc., 312-CI filtered at 1,000 Hz) while being viewed with an inverted microscope at 320x magnification. Sarcomere length was determined by spatial Fourier transformation. Maximal force (F_max_) and passive force (F_pass_) of sarcomeres were measured at high [Ca^2+^] and low [Ca^2+^] (pCa 4.5 and 9.0, respectively). Force-[Ca^2+^] curves were constructed at various submaximal [Ca^2+^] and are shown as relative forces to F_max_. Myofilament Ca^2+^-sensitivity was measured as the [Ca^2+^] needed to achieve 50% of F_max_ (EC_50_). Length-dependent activation was measured as the shift in EC_50_ (ΔEC_50_) at a sarcomere length of 1.8 and 2.2 μm. In order to correct for differences in protein kinase A (PKA)-mediated phosphorylation of sarcomeric proteins, membrane permeabilized cardiomyocytes were incubated with 80 μl 1 unit/μl PKA (P5511, Sigma) and 0.006 mM cAMP (Sigma) in isolation relax solution at 20°C for 40 min prior to measurement of force development.

### Titin expression and cardiac troponin I phosphorylation

Titin isoforms were separated on a 1% w/v agarose gel and stained with SYPRO Ruby protein gel stain (Invitrogen) as previously described (Warren et al., 2003). All samples were measured in triplicate and the average of triplicate measurements per sample is shown. Phosphorylation of cardiac troponin I (cTnI) was assessed as previously described (Zaremba et al., 2007; Najafi et al., 2015). Non-, mono-, and bis-phosphorylated cTnI (Pierce, MA1-22700) were separated by polyacrylamide bound Mn^2+^-phos-tag gel electrophoresis western blotting as previously described (Najafi et al., 2015). Images were captured with AI600 (GE Healthcare Life Sciences) and analyzed with ImageQuantTL software (GE Healthcare Life Sciences). Raw blot images of the representative images can be seen as Supplementary Material online.

### HSP27, HSP70, LC3B-I/II, and p62 expression

For HSP70 and HSP27 analysis proteins were separated on pre-cast 10% criterion gels (BioRad) and membranes were incubated with HSP70 antibody (Enzo) or HSP27 antibody (Cell Signaling), and GAPDH antibody (Cell signaling) to correct for loading differences. For LC3B-I and LC3B-II analysis proteins were separated on pre-cast 8–16% gradient TGX gels (BioRad) and membranes were incubated with LC3B-I/II antibody. Analysis of p62 expression was performed by separating proteins on a 12% acrylamide gel and the membrane was cut in two parts. The upper part was incubated with p62 antibody (Cell signaling) and the lower part with GAPDH antibody. Data from different blots was combined by loading the same sample on each gel and normalizing all for this sample. Images were captured with AI600 (GE Healthcare Life Sciences) and analyzed with ImageQuantTL software (GE Healthcare Life Sciences). Raw blot images of the representative images can be seen as Supplementary Material online.

### Electron microscopy

Cardiac tissue of pediatric CM and control samples were studied with transmission electron microscopy. Myocardium was fixed in 2% paraformaldehyde and 2.5% glutaraldehyde in 0.1M phosphate buffer (pH 7.4), embedded in Epon and cut in 70 nm sections. The sections were mounted onto formvar-coated copper grids and stained with a 5% solution of uranyl acetate, followed by Reynold's lead citrate. Sections were viewed with Philips CM100 Transmission Electron Microscope. The myofibril density was determined with ImageJ software and expressed as the percentage of whole cell area in that picture. The nucleus was excluded from analysis. For each sample 2–7 different images were analyzed in order to determine average myofibril density. Maximal and passive forces were divided by the average myofibril density of the corresponding sample.

### Statistics

Graphpad Prism v7 software was used for statistical analysis. Means were compared between groups with *T*-test if data was normally distributed. Maximal force data was not normally distributed and therefore means were compared with a Mann-Whitney test. A *p* < 0.05 was considered to represent a statistically significantly difference between groups. *N* = number of samples, *n* = number of cardiomyocytes measured. Correlation analysis was performed with linear regression analysis in Graphpad Prism v7 software. A correlation is considered significant if the slope of the linear regression is significantly not zero (*p* < 0.05).

### Ethics statement

This study was approved by the local ethics board of the Erasmus Medical Center (protocol number MEC-2015-233) and written informed consent of patients and/or parents was obtained. Samples were obtained during cardiac transplantation. As we do not have access to control cardiac tissue from age-matched individuals, 18 control samples were used from explanted Left ventricular (LV) heart tissue of healthy donors (age range 21–65 years old). These healthy donors are people who died from a non-cardiac cause, typically motor vehicle accidents and were acquired from the University of Sydney, Australia, with the ethical approval of the Human Research Ethics Committee #2012/2814. The codes of control samples are: 6.034, 8.004, 5.086, 3.141, 3.164, 4.049, 4.104, 7.040, 6.020, 5.128, 3.160, 6.008, 7.054, 7.044, 6.056, 3.112, 6.042, and 3.162. All samples were stored in liquid nitrogen or at −80°C until use.

## Results

### Patient characteristics

Heart tissue was obtained from 9 pediatric patients diagnosed with DCM and 2 pediatric patients diagnosed with non-compaction cardiomyopathy during cardiac transplantation. Z-scores are commonly used to define growth of the heart during development and are used to distinguish between physiological and pathological changes in pediatric patients (Chubb and Simpson, 2012). Patient characteristics are shown in Table 1. LV end systolic diameter (LVESD) and LV end diastolic diameter (LVEDD) were increased and LV posterior wall systole (LVPWs) and LV posterior wall diastole (LVPWd) were decreased, all in line with the dilated phenotype and diagnosis of DCM.

**Table 1:** Patient characteristics.

	**Controls (*N* = 18)**	**Pediatric CM (*N* = 11)**
Age	44.1 ± 3.1 years (*N* = 18)	10.5 ± 1.3 years (*N* = 11)
Sex (% male)	55.6 (*N* = 18)	45.5 (*N* = 11)
Time between echo and HTX		15 days (11–64)
LVEDD Z-score		7.9 ± 1.0 (*N* = 11)
LVESD Z-score		11.7 ± 1.0 (*N* = 11)
LVPWd Z-score		−0.8 ± 0.5 (*N* = 11)
LVPWs Z-score		−3.4 ± 0.5 (*N* = 8)
FS % at presentation		8 (7–10) (*N* = 8)
FS % at HTx		15 (8–19) (*N* = 8)
NT-pro-BNP at HTx		483 (206–1,034) (*N* = 10)

*Age, LVEDD, LVESD, LVPWd, LVPWs are shown as mean ±SEM. Time between echo and HTX, FS%, and NT-pro-BNP (pmol/L) are shown as median (interquartile range). HTX, Cardiac transplantation*.

### Hypocontractility and increased myofilament Ca^2+^-sensitivity in pediatric CM compared to controls

**Figure 1:**
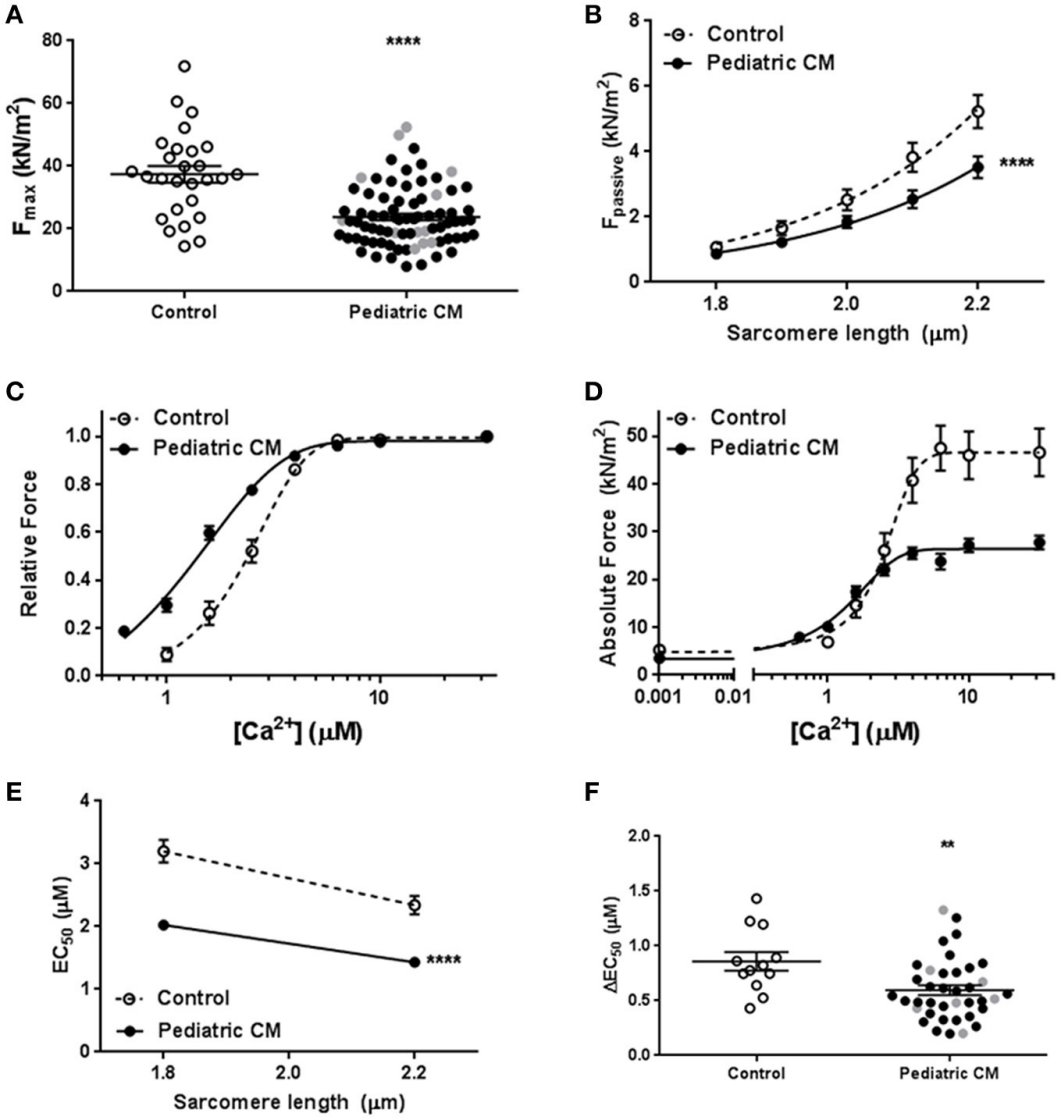
Baseline characteristics. **(A)** Maximal force was significantly lower in pediatric CM (23.8 ± 1.1, *N* = 11, *n* = 78) compared to controls (37.4 ± 2.6, *N* = 6, *n* = 27, *p* < 0.0001). **(B)** F_pass_ was significantly lower in pediatric CM (*N* = 11, *n* = 40) compared to controls (*N* = 10, *n* = 29, *p* < 0.0001) over a range of sarcomere lengths. **(C)** A leftward shift of the relative force vs. [Ca^2+^] indicates higher myofilament Ca^2+^-sensitivity in pediatric CM (*N* = 11, *n* = 38) compared to controls (*N* = 5, *n* = 12, *p* < 0.0001). **(D)** The absolute force development over a range of [Ca^2+^] showed pediatric CM have impaired maximal force development at saturating [Ca^2+^], increased force development at lower [Ca^2+^] and a decreased F_pass._ **(E)** Ca^2+^-sensitivity was significantly higher at sarcomere lengths of 1.8 and 2.2 μm in pediatric CM (*N* = 11, *n* = 38) compared to controls (*N* = 5, *n* = 12, *p* < 0.0001). **(F)** Length-dependent activation, measured as ΔEC_50_ was significantly lower in pediatric CM (0.60 ± 0.05, *N* = 11, *n* = 38) compared to controls (0.86 ± 0.09, *N* = 5, *n* = 12, *p* = 0.007). *N*, number of samples; *n*, number of cardiomyocytes measured. Measurements obtained from samples derived from patients with non-compaction cardiomyopathy are indicated in gray **(A,F)**. ^**^p < 0.01, ^****^p < 0.0001 vs. controls.

Measurements in single cardiomyocytes revealed significantly lower F_max_ (Figure 1A) and F_pass_ (Figure 1B) in pediatric CM compared to controls. A leftward shift of the force-[Ca^2+^] curve indicated an increased myofilament Ca^2+^-sensitivity in pediatric CM compared to controls (Figure 1C). The combination of reduced F_max_, reduced F_pass_, and increased Ca^2+^-sensitivity of myofilaments results in lower force development at high (saturating) [Ca^2+^], lower force at very low [Ca^2+^], and higher force development at intermediate [Ca^2+^], respectively (Figure 1D). The increase in myofilament Ca^2+^-sensitivity was evident at both sarcomere lengths (1.8 and 2.2 μm; Figure 1E). The slope of the curve in Figure 1E represents the ability to increase contractility upon stretch and is called length-dependent activation. Length-dependent activation is the cellular basis of the Frank-Starling mechanism. Length-dependent activation is often expressed as the shift in calcium sensitivity upon stretch. Pediatric CM cardiomyocytes showed an impaired length-dependent activation depicted as a decreased ΔEC_50_ compared to control cardiomyocytes (Figure 1F).

### Titin isoform composition variation in pediatric CM has limited effect on contractility

**Figure 2:**
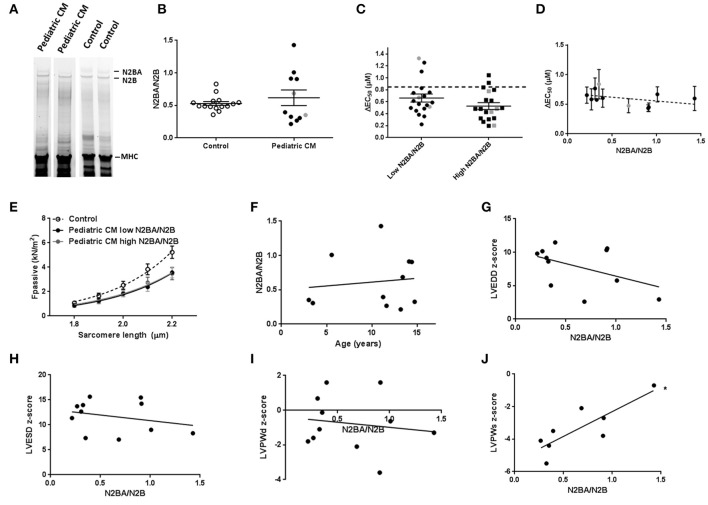
Titin isoform composition has limited effect on contractility in pediatric CM. **(A)** Separation of titin N2BA and N2B with gel electrophoresis. **(B)** N2BA/N2B ratio was not significantly different between pediatric CM (0.62 ± 0.12, *N* = 11) and controls (0.53 ± 0.03, *N* = 15). **(C)** Length-dependent activation was mostly impaired in pediatric CM patients who had higher N2BA/N2B ratio (N2BA/N2B > 0.65, *N* = 5, *n* = 19) compared to pediatric CM patients who had lower N2BA/N2B ratio (N2BA/N2B < 0.4, *N* = 6, *n* = 19). Dotted line indicates control values. **(D)** Mean ΔEC_50_ per sample plotted against the N2BA/N2B ratio did not show a significant correlation between ΔEC_50_ and N2BA/N2B. **(E)** There was no difference in F_pass_ between pediatric CM patients who had higher N2BA/N2B ratio (N2BA/N2B > 0.65, *N* = 5, *n* = 18) and pediatric CM patients who had lower N2BA/N2B ratio (N2BA/N2B < 0.4, *N* = 6, *n* = 22). **(F)** N2BA/N2B ratio was not significantly related to age. **(G)** No significant correlation was found between N2BA/N2B and LVEDD z-score. **(H)** No significant correlation was found between N2BA/N2B and LVESD z-score. **(I)** No significant correlation was found between N2BA/N2B and LVPWd z-score. **(J)** A significant correlation was found between N2BA/N2B ratio and LVPWs z-score (*p* < 0.05). *N*, number of samples; *n*, number of cardiomyocytes measured. Measurements obtained from samples derived from patients with non-compaction cardiomyopathy are indicated in gray **(B–D)**.

A shift toward more N2BA titin isoform has been shown to lower F_pass_ (Granzier and Irving, 1995; Makarenko et al., 2004; Nagueh et al., 2004) and reduce length-dependent activation (Fukuda et al., 2003). Analysis of titin isoform composition did not reveal a difference in the N2BA/N2B ratio between pediatric CM and controls (Figures 2A,B). However, a wide variation in titin isoform composition was observed in the pediatric CM group (Figure 2B). Since titin isoform composition has been shown to affect length-dependent activation we divided samples into a group that showed a high N2BA/N2B ratio (N2BA/N2B > 0.65) and a low N2BA/N2B ratio (N2BA/N2B < 0.4). Both groups showed lower ΔEC_50_ compared to controls and there was no significant difference in ΔEC_50_ between both pediatric CM groups. However, the group of samples with a high N2BA/N2B ratio showed a slightly but non-significantly greater reduction in ΔEC_50_ than the group of samples with a low N2BA/N2B ratio (Figure 2C). In addition, ΔEC_50_ did not significantly correlate with the N2BA/N2B ratio (Figure 2D). Accordingly, F_pass_ was reduced to the same extent in the groups of pediatric CM samples with a relatively high N2BA/N2B ratio (N2BA/N2B > 0.65) and a low N2BA/N2B ratio (N2BA/N2B < 0.4) (Figure 2E). This indicates that other factors underlie the reduction of F_pass_.

The wide spread in age of our patient population was not responsible for the spread of titin isoform composition since no correlation was found between age and N2BA/N2B ratio (Figure 2F). We observed a significant correlation between N2BA/N2B and LVPWs (Figure 2J) in which a high N2BA/N2B ratio was associated with a less negative LVPWs z-score. This implies that patients with more compliant titin isoform had a smaller reduction in systolic LV wall thickness. No significant correlation was found between N2BA/N2B and LVEDD z-score (Figure 2G), LVESD z-score (Figure 2H) or LVPWd z-score (Figure 2I).

### Lower phosphorylation of cTnI in pediatric CM compared to controls

**Figure 3:**
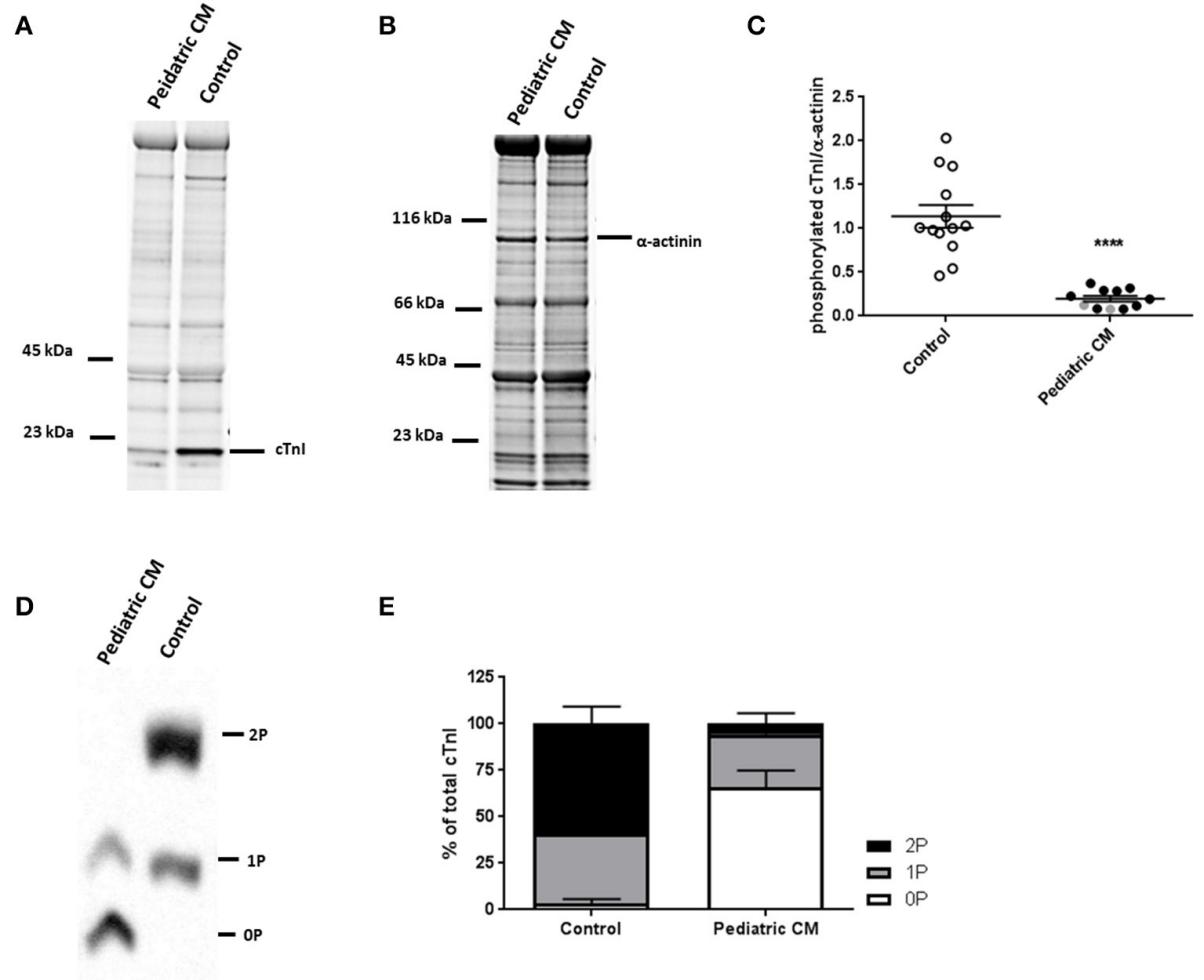
Hypophosphoryalation in pediatric CM compared to controls. **(A)** ProQ staining identifying phosphorylated proteins of pediatric CM and control samples. **(B)** Corresponding SYPRO staining identifying proteins of pediatric CM and control samples. **(C)** cTnI phosphorylation was significantly lower in pediatric CM (*N* = 11) compared to controls (*N* = 13, *p* < 0.0001). **(D)** Phostag showed separation of non-, mono-, and bisphosphorylated cTnI. **(E)** While controls (*N* = 11) showed predominantly mono- and bisphosphorylated cTnI, pediatric CM samples (*N* = 11) showed mostly non-phosphorylated cTnI. Measurements obtained from samples derived from patients with non-compaction cardiomyopathy are indicated in gray. ^****^p < 0.0001 vs. controls.

**Figure 4:**
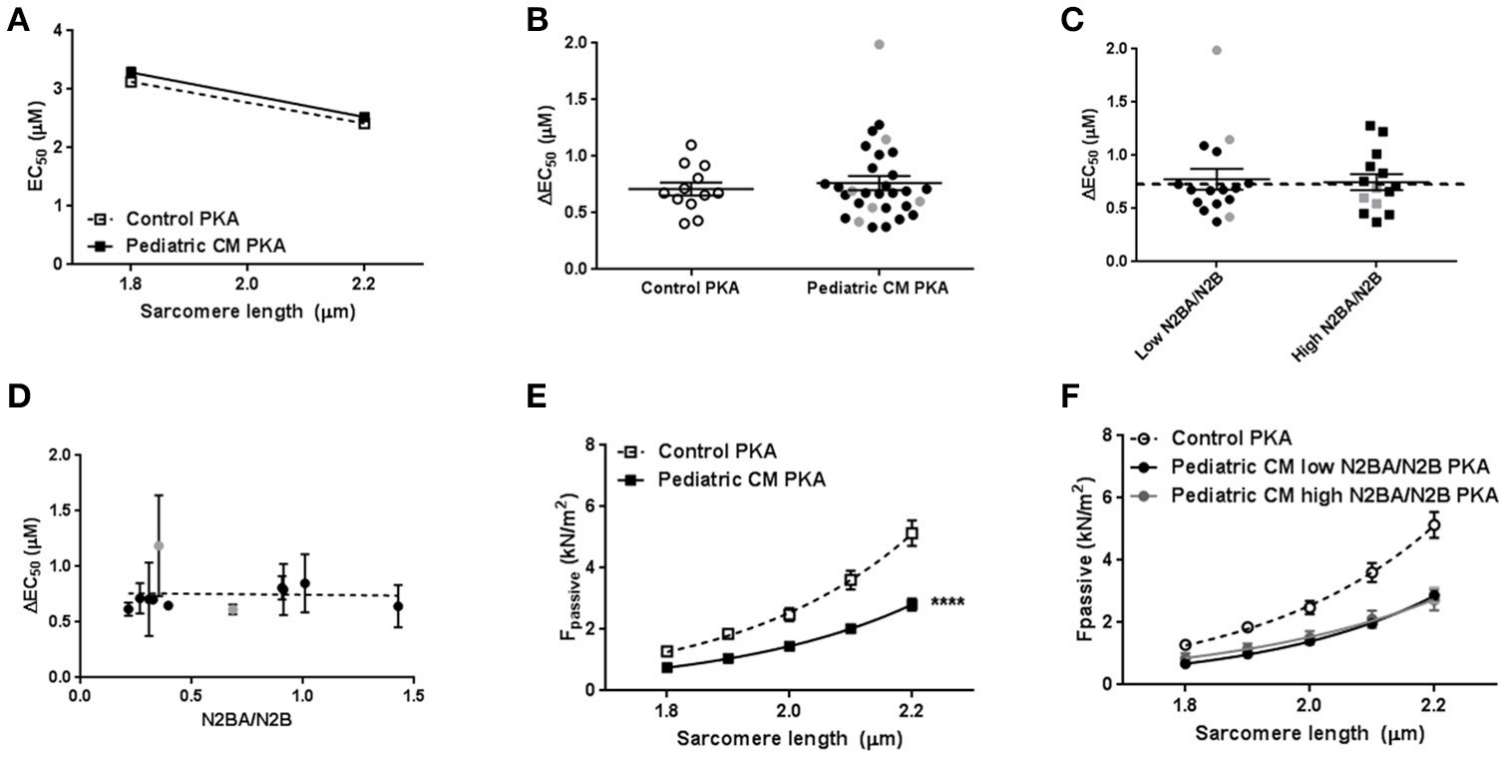
Restoration of sarcomere function after incubation with exogenous PKA. **(A)** Exogenous PKA restored myofilament Ca^2+^-sensitivity in pediatric CM (*N* = 11, *n* = 30) to control (*n* = 5, *n* = 12) values. **(B)** Exogenous PKA eliminated the difference in length-dependent activation (ΔEC_50_) between pediatric CM patients with higher N2BA/N2B ratio (N2BA/N2B > 0.65, *N* = 5, *n* = 14) and pediatric CM patients who had lower N2BA/N2B ratio (N2BA/N2B < 0.4, *N* = 6, *n* = 16) (shown relative to control value). Dotted line indicates control values. **(C)** There was no difference between patients with high or low N2BA/N2B ratio with respect to ΔEC_50_ after incubation with exogenous PKA. **(D)** Mean ΔEC50 per sample measured after incubation with exogenous PKA plotted against the N2BA/N2B ratio did not show a significant correlation between ΔEC50 and N2BA/N2B. **(E)** Exogenous PKA did not affect F_pass_ in controls or pediatric CM. F_pass_ remained significantly lower in pediatric CM compared to controls. **(F)** There was no difference in F_pass_ after incubation with exogenous PKA between pediatric CM patients who had higher N2BA/N2B ratio (N2BA/N2B > 0.65, *N* = 5, *n* = 13) and pediatric CM patients who had lowerN2BA/N2B ratio (N2BA/N2B < 0.4, *N* = 6, *n* = 18). *N*, number of samples; *n*, number of cardiomyocytes measured. Measurements obtained from samples derived from patients with non-compaction cardiomyopathy are indicated in gray **(B–D)**.

Phosphorylation of cTnI is reduced in various forms of adult heart failure and causes increased myofilament Ca^2+^-sensitivity (van Dijk et al., 2009; Sequeira et al., 2013; Beqqali et al., 2016; Bollen et al., 2017b). In line with published data in adult DCM (Wijnker et al., 2014; Beqqali et al., 2016; Bollen et al., 2017b), phosphorylation of cTnI was significantly lower in pediatric CM compared to controls (Figures 3A–C). PhosTag analysis showed separation of non-, mono- and bisphosphorylated cTnI (Figure 3D). While controls showed predominantly bisphosphorylated cTnI, in pediatric CM patients the non-phosphorylated cTnI was more prevalent (Figure 3E). In order to confirm that cTnI hypophosphorylation causes the high myofilament Ca^2+^-sensitivity in pediatric CM samples, force measurements were repeated after incubation with exogenous PKA which phosphorylates cTnI. Both myofilament Ca^2+^-sensitivity and length-dependent activation normalized to control values upon incubation with exogenous PKA (Figures 4A,B). With normalized phosphorylation status of cTnI, there was no difference in ΔEC_50_ between the group of pediatric CM samples with a low N2BA/N2B ratio and a high N2BA/N2B ratio (Figure 4C). Also after incubation with exogenous PKA, ΔEC50 did not significantly correlate with N2BA/N2B ratio (Figure 4D). The slope of the curve which indicates the strength of the correlation, was small and non-significant at baseline (Figure 2D) and was even smaller after incubation with exogenous PKA (Figure 4D). This implies that any effect of N2BA/N2B ratio on length-dependent activation was diminished upon incubation with exogenous PKA. F_pass_ also remained low after incubation with exogenous PKA (Figure 4E). In addition, no difference in F_pass_ was observed between PKA-treated groups of pediatric CM samples with different N2BA/N2B ratios (Figure 4F).

### Decreased myofibril density underlies the hypocontractility in pediatric CM

**Figure 5:**
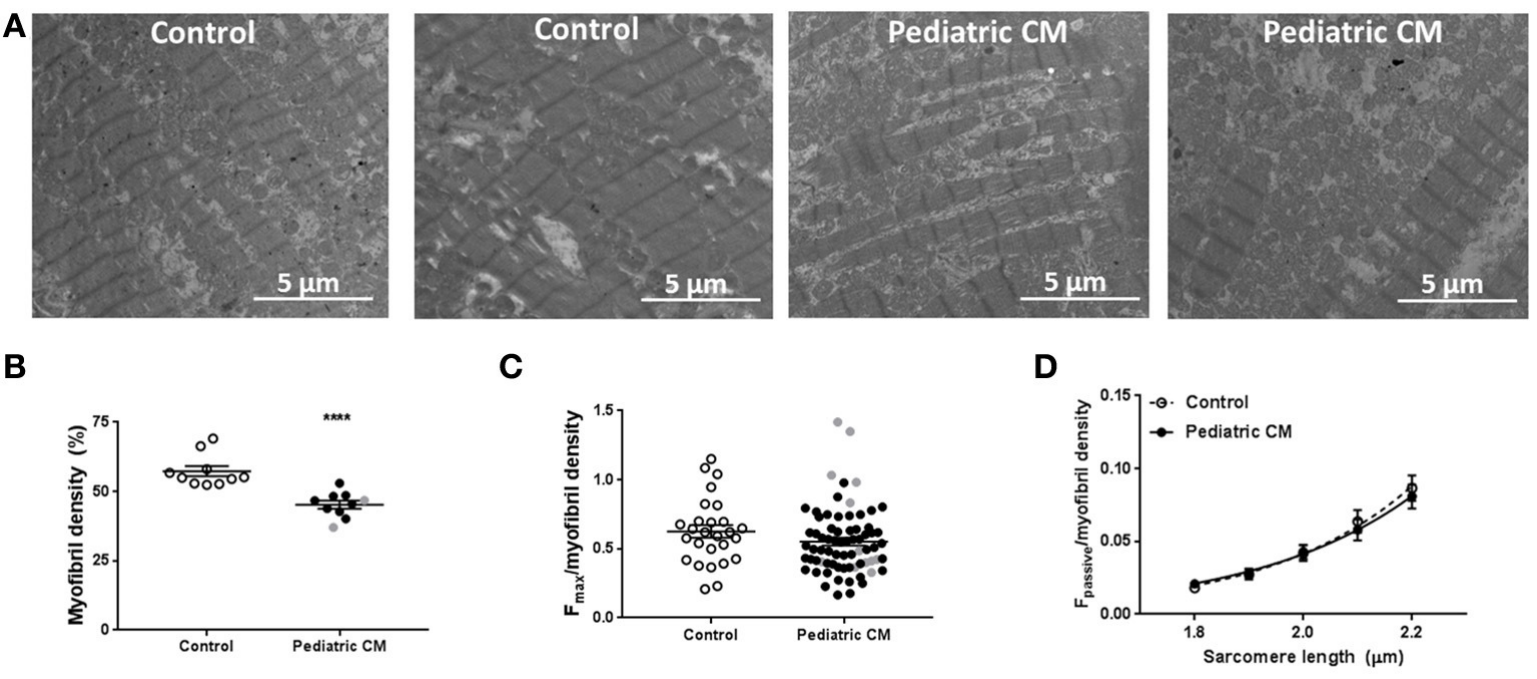
Myofibril density is decreased in pediatric CM. **(A)** Electron microscopy images of 2 control samples and 2 pediatric CM samples. **(B)** Myofibril density was significantly lower (*P* < 0.0001) in pediatric CM (*N* = 10, 45.3 ± 1.4%) compared to controls (*N* = 10, 57.3 ± 1.8%). **(C)** F_max_ normalized for myofibril density of corresponding sample did not differ significantly between pediatric CM (*N* = 10, *n* = 71) and controls (*N* = 6, *n* = 27). **(D)** F_pass_ normalized for myofibril density of corresponding sample was not significantly different between pediatric CM (*N* = 10, *n* = 37) and controls (*N* = 9, *n* = 25). *N*, number of samples; *n*, number of cardiomyocytes measured. Measurements obtained from samples derived from patients with non-compaction cardiomyopathy are indicated in gray. ^****^p < 0.0001 vs. controls.

As titin isoform composition was ruled out as cause of the observed decrease in F_pass_ and F_max_, we determined myofibril density with transmission electron microscopy. We observed a significantly lower myofibril density in pediatric CM compared to controls (Figures 5A,B). Correction of F_max_ for myofibril density eliminated the difference between pediatric CM and controls indicating the hypocontractility was due to myolysis or the inability to create sufficient myofibrils (Figure 5C). Also F_pass_ of pediatric CM cardiomyocytes was restored to control values after correction for myofibril density (Figure 5D).

### Protein quality control system is unaltered in pediatric CM

**Figure 6:**
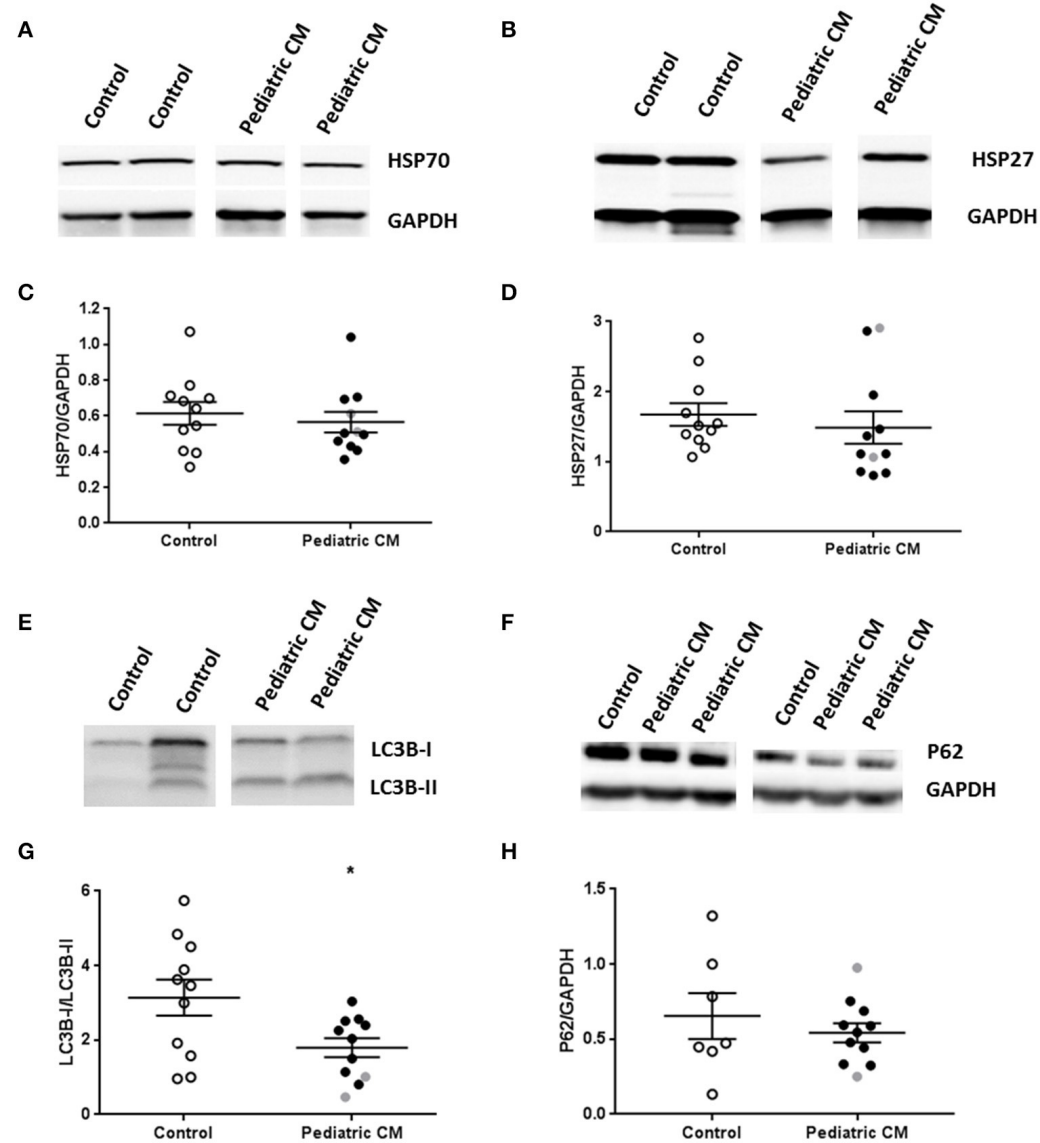
Protein quality control system in pediatric CM. **(A)** Representative blot images for HSP27 expression. **(B)** Representative blot images for HSP70 expression. **(C)** Expression of HSP27 was not altered in pediatric CM (*N* = 11) compared to controls (*N* = 11). **(D)** Expression of HSP70 was not altered in pediatric CM (*N* = 11) compared to controls (*N* = 11). **(E)** Representative blot images for LC3BI and LC3B-II expression. **(G)** Expression of LC3B-I/LC3-BII was significantly (*p* < 0.05) reduced in pediatric CM (*N* = 11) compared to controls (*N* = 11). **(F)** Representative blot images for p62 expression. **(H)** Expression of p62 was not altered in pediatric CM (*N* = 11) compared to controls (*N* = 7). GAPDH was used a loading control in the HSP27, HSP70, and p62 blots. Measurements obtained from samples derived from patients with non-compaction cardiomyopathy are indicated in gray. ^*^p < 0.05, vs. controls.

We then studied whether changes in the protein quality control system occurred, which may underlie reduced myofibril density. Heat shock proteins (HSPs) are upregulated in response to cellular stress in order to prevent protein denaturation, and aid in refolding of misfolded proteins. We did not find an induction of HSP70 (Figures 6A,C) or the cytoskeletal HSP27 (Figures 6B,D) in our pediatric CM group compared to controls. We did find a significantly decreased LC3B1/LC3B-II ratio (Figures 6E,G), which implies an induction of autophagy. However, p62, another autophagy marker, was not upregulated in pediatric CM compared to controls (Figures 6F,H).

## Discussion

Characterization of end-stage pediatric CM myocardium revealed reduced active and passive cardiomyocyte force development, high myofilament Ca^2+^-sensitivity and a blunted length-dependent activation compared to adult non-failing controls. High myofilament Ca^2+^-sensitivity and blunted length-dependent activation were explained by low PKA-mediated phosphorylation of cTnI, which is a general feature observed in cardiac disease. We showed that the decrease in F_max_ and F_pass_ was due to decreased myofibril density.

### No indications for alterations in protein quality control system in pediatric CM

We observed a decrease in myofibril density which was causal to the hypocontractile cellular phenotype in end-stage pediatric CM compared to adult non-failing controls. A reduction in myofibril density and subsequent decreased maximal force development has been observed in adult cardiomyopathy due to specific mutations (Witjas-Paalberends et al., 2013; Bollen et al., 2017b; Hoorntje et al., 2017). Maximal force development was not reduced in adult idiopathic DCM or in DCM caused by cardiac troponin I and T mutations (Bollen et al., 2017a,b). Even though these studies did not measure myofibril density, these findings imply that the hypocontractility observed in pediatric CM samples is not a general DCM feature.

It has been reported that myofibril density is lower during prenatal development and increases in neonatal cardiac tissue (Racca et al., 2016). Force development was also lower in prenatal and neonatal myofibrils compared to adult myofibrils (Racca et al., 2016). To our knowledge no reports about myofibril density in healthy individuals in the age range we studied are available. The pediatric samples we studied were derived from patients with an age range between 3 and 15 years old. We did not find an age-dependent change in myofibril density in our pediatric patients. This implies that it is unlikely that the reduced myofibril density we observed in the pediatric CM samples compared to adult control samples is a reflection of a lower myofibril density at young age.

Reduced HSP expression has been shown to increase cardiac damage after brief ischemia, and pretreatment with heat to induce HSPs reduced cardiac damage after infarction. However, in conditions of continuous stress as is the case in DCM, the HSP responses are less clear. HSP27 has been shown to be upregulated in adult DCM and not in ischemic heart disease (Knowlton et al., 1998). There are conflicting reports about HSP70 in adult DCM ranging from no change (Knowlton et al., 1998), to an increased expression (Barrans et al., 2002). HSPs may lose their responsiveness in conditions of continuous cardiac stress. We did not find an induction of heat shock response, and we also did not find more autophagosomes in pediatric CM compared to controls. Future studies are warranted to reveal if reduced myofibril density in pediatric CM is due to an inability of cardiomyocytes to increase myofibril synthesis and/or is caused by increased myofibril degradation.

### Low PKA-mediated phosphorylation, high myofilament Ca^2+^-sensitivity and blunted length-dependent activation

In line with what has been found in adult DCM patients (van Dijk et al., 2009; Bollen et al., 2017b), we observed decreased cTnI phosphorylation which caused an increase in Ca^2+^-sensitivity in end-stage pediatric CM patients compared to controls. Hypophosphorylation of cTnI has been shown to occur in various forms of heart failure. It is likely due to desensitization of the β-adrenergic receptor signaling pathway and subsequent decrease in PKA-mediated phosphorylation (Harding et al., 1994). Hypophosphorylation of cTnI has been shown to underlie a blunted length-dependent activation (Fukuda et al., 2009; Wijnker et al., 2013, 2014). Treatment of cardiomyocytes with exogenous PKA corrected both Ca^2+^-sensitivity of myofilaments and length-dependent activation, independent of the titin isoform composition present in the heart.

In early postnatal life a slow skeletal isoform of TnI (ssTnI) is present in the heart and this isoform has been shown to increase Ca^2+^-sensitivity (Fentzke et al., 1999). However, the ssTnI isoform can no longer be detected at day 14 after birth in mice and has been replaced by cTnI (Huang et al., 1999). It has been shown that ssTnI is not responsive to PKA treatment (Fentzke et al., 1999). Since we showed that blunted length-dependent activation and high Ca^2+^-sensitivity could be normalized to adult non-failing controls with exogenous PKA treatment this implies that ssTnI is either not present or at such low levels in the studied pediatric CM samples that its effect on Ca^2+^-sensitivity and length-dependent activation can be neglected.

### Titin isoform composition is highly diverse in pediatric CM

Titin is composed of two isoforms, a compliant N2BA isoform and a stiff N2B isoform. Various animal models, including pigs, rats, mice, and rabbits, have shown that a very compliant fetal isoform is expressed early in life and disappears quickly after birth (Lahmers et al., 2004). In pigs, which heart is often considered to represent a human heart better than rodents, this fetal isoform is completely replaced by the adult isoforms at 180 days after birth. An increase in N2BA/N2B ratio has been reported in various forms of heart failure including adult DCM (Makarenko et al., 2004; Nagueh et al., 2004; Bollen et al., 2017b) and is considered a general hallmark of DCM. However, in our pediatric CM study population we observed a wide variation of N2BA/N2B ratios: 5 patients showed a higher N2BA/N2B ratio, while 6 patients showed a normal or even lower N2BA/N2B ratio compared to controls. The shift in titin isoform composition in DCM is not due to re-expression of the fetal isoform which is of a significantly larger size (Bollen et al., 2017a). Re-expression of this larger titin isoform has been observed in DCM, but only in samples or animal models in which a mutation was found or introduced in the gene encoding the splicing regulator of titin: RBM20 (Methawasin et al., 2014; Beqqali et al., 2016). On average we observed no significant change in titin isoform composition in pediatric CM compared to controls. The pediatric CM samples in this study did not differ in location of the titin isoforms in the gels compared to adult non-failing controls. This implies that the fetal isoform is not present as this isoform should be located at a much higher position in the gel. In addition, we did not find a significant correlation between age and N2BA/N2B ratio which implies that the wide variation in N2BA/N2B ratio we observed was not due to the spread in age within the pediatric CM group or biased by ongoing developmental changes in childhood.

An increase in N2BA titin has been shown to cause a blunted length-dependent activation in animal models,(Fukuda et al., 2003, 2009; Inoue et al., 2013; Kobirumaki-Shimozawa et al., 2014) while we only observed a modest effect of N2BA on length-dependent activation of myofilaments in human pediatric CM samples. The length-dependent increase in myofilament Ca^2+^-sensitivity was slightly, though not significantly lower in pediatric CM samples with a high N2BA/N2B ratio compared to samples with a low N2BA/N2B ratio. There was no significant difference in ΔEC_50_ between pediatric CM samples with a low or high N2BA/N2B ratio and both groups showed a reduced ΔEC_50_ compared to controls. This implies that the blunted length-dependent activation observed in pediatric CM samples was not solely caused by samples with a high N2BA/N2B ratio. Length-dependent activation of myofilaments was normalized in all pediatric CM samples to control values after incubation with PKA which indicates that the increase in compliant titin only contributes to the impairment of length-dependent activation of myofilaments when cTnI is hypophosphorylated. This is in line with what has been reported in adult DCM (Beqqali et al., 2016; Bollen et al., 2017b). While increased N2BA/N2B ratio has been associated with impaired systolic function, a low N2BA/N2B ratio is associated with improved diastolic function. A positive correlation between N2BA/N2B and peak oxygen consumption, a measure for exercise tolerance, in DCM patients has been found (Nagueh et al., 2004). We observed that a high N2BA/N2B ratio coincided with a smaller reduction in LV wall thickness during systole (LVPWs). An increase in N2BA/N2B ratio may represent a compensatory mechanism in order to cope with altered cardiac stress. The correlation between increased N2BA/N2B ratio and a smaller reduction in LVPWs may imply that an increase in N2BA/N2B ratio has a limited impact on sarcomere function in end-stage pediatric CM. This is in line with the moderate effect of increased N2BA/N2B on the impairment of length-dependent activation. However, it should be stated that all studied heart tissue was derived from end-stage pediatric CM patients and therefore might suffer from severe cardiac remodeling. It might be possible that N2BA/N2B aids in this remodeling in a positive way by creating more flexibility in the sarcomeric structure to function under overstretched conditions at the initial disease stage.

### Cardiac remodeling: friend or foe?

The increase in compliant titin might not have a direct causal role in disease pathogenesis, but may rather represent an adaptive response in order to cope with altered cardiac demand. The inability of pediatric CM patients to upregulate N2BA expression might be a reflection of their limited capability to adapt to altered cardiac demands. This is in line with Patel et al. who recently published limited adverse remodeling in pediatric CM patients compared to adult onset DCM (Patel et al., 2017). They showed that hypertrophy and perivascular and interstitial fibrosis was increased in adult DCM but not in pediatric CM patients compared age-matched controls. In addition, they showed sarcomere thickness was increased in adult DCM, but not in pediatric CM (Patel et al., 2017). Limited cardiac remodeling in pediatric CM patients might hamper the hearts to cope with altered cardiac demands. If the limited adaptive capabilities of the heart are indeed causal to the early and progressive disease onset warrants further research.

### Limitations

We have compared end-stage pediatric DCM patients with healthy adult controls since acquisition of healthy control tissue of children is near impossible. However, we did not find any correlations between age and protein expression or sarcomere function. Therefore we believe the observed changes are not related to difference in age.

Three patients were supported with a ventricular assist device (VAD) prior to transplantation. The duration of VAD support was short (1–2 months). We did not see a difference in any parameter between patients that were supported with a VAD and patients that were not supported prior to transplantation. However, we cannot exclude that VAD also affected cardiac remodeling.

## Conclusion

In summary, we show that end-stage pediatric CM patients harbor similar changes in protein modifications and sarcomeric function compared to adult DCM. Hypophoshorylation of cTnI, most likely due to secondary disease remodeling and desensitized β-adrenergic receptor signaling, led to increased Ca^2+^-sensitivity and blunted length-dependent activation of myofilaments. We did not find the consistent upregulation of compliant N2BA titin isoform that has been observed in adult DCM. Increased N2BA/N2B ratio was significantly related to a lower LVPWs z-score. The limited cardiac remodeling in pediatric CM patients, illustrated in this study by the limited shift in titin isoform composition, might have hampered the ability to cope with altered cardiac demands and might have contributed to their early disease onset and progression. We found cardiomyocyte hypocontractility which was caused by a significant decrease in myofibril density. The severe reduction in force generating capacity of cardiomyocytes may underlie the fast progression of cardiac disease in pediatric patients.

## Author contributions

IB performed and analyzed the contractile force experiments, titin isoform composition analysis, imaged samples with TEM, analyzed myofibril density, performed overall data interpretation, and manuscript production. MvdM and MD acquired patient samples and clinical data and assisted with data interpretation and manuscript production. KdG performed and analyzed protein phosphorylation experiments. DK and JvdV were involved in overall study design, data interpretation and manuscript production. All authors critically revised the manuscript and approved it for publication.

### Conflict of interest statement

The authors declare that the research was conducted in the absence of any commercial or financial relationships that could be construed as a potential conflict of interest.
