# Supplementary figures and images for: Cardiomyocyte Hypocontractility and Reduced Myofibril Density in End-Stage Pediatric Cardiomyopathy
Source: Front Physiol. 2017 Dec 22;8:1103. doi: 10.3389/fphys.2017.01103 (PMC5743800; doi:10.3389/fphys.2017.01103)

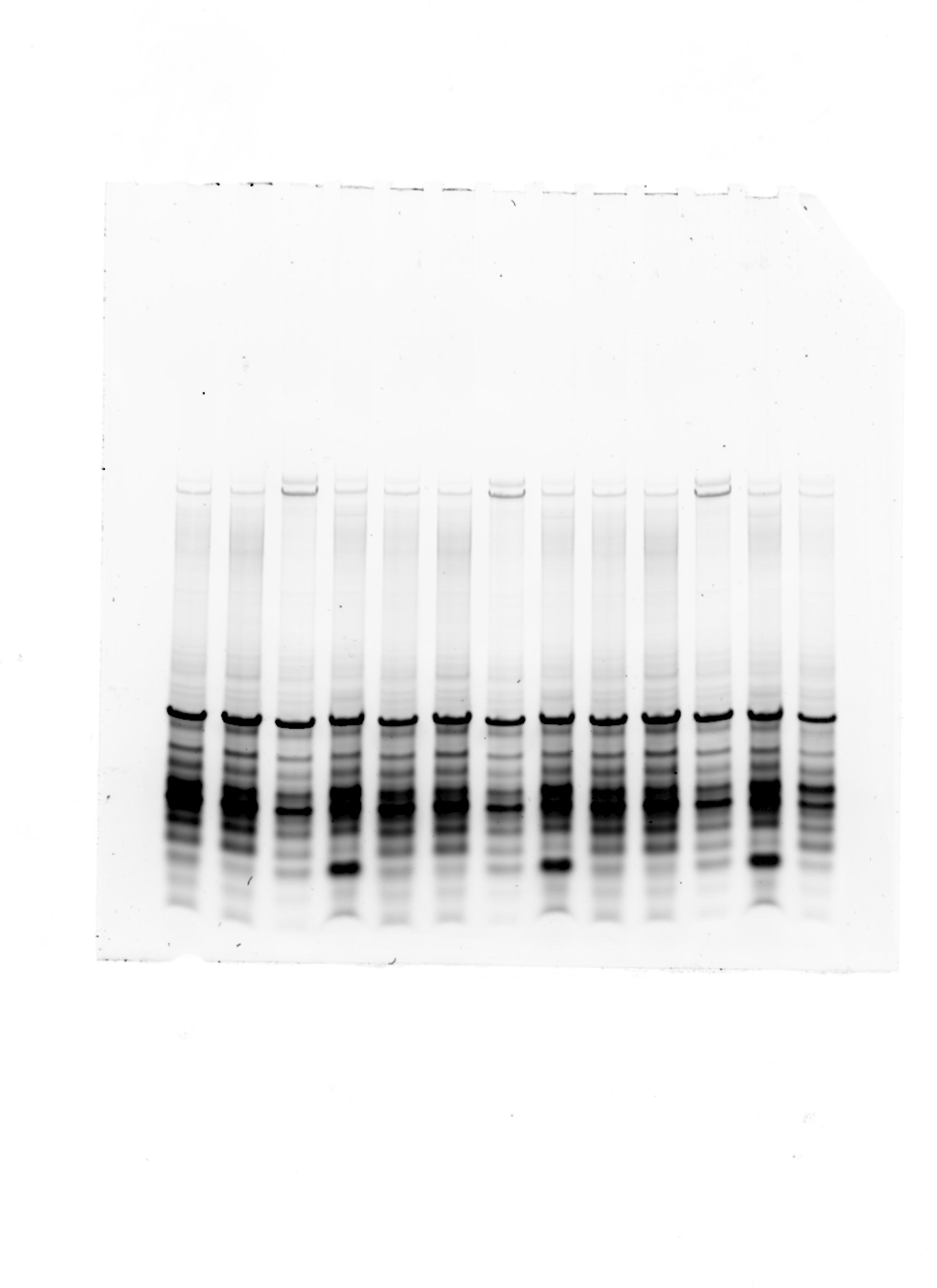

Supplement: Supplementary file 1 [file Image1.JPEG]

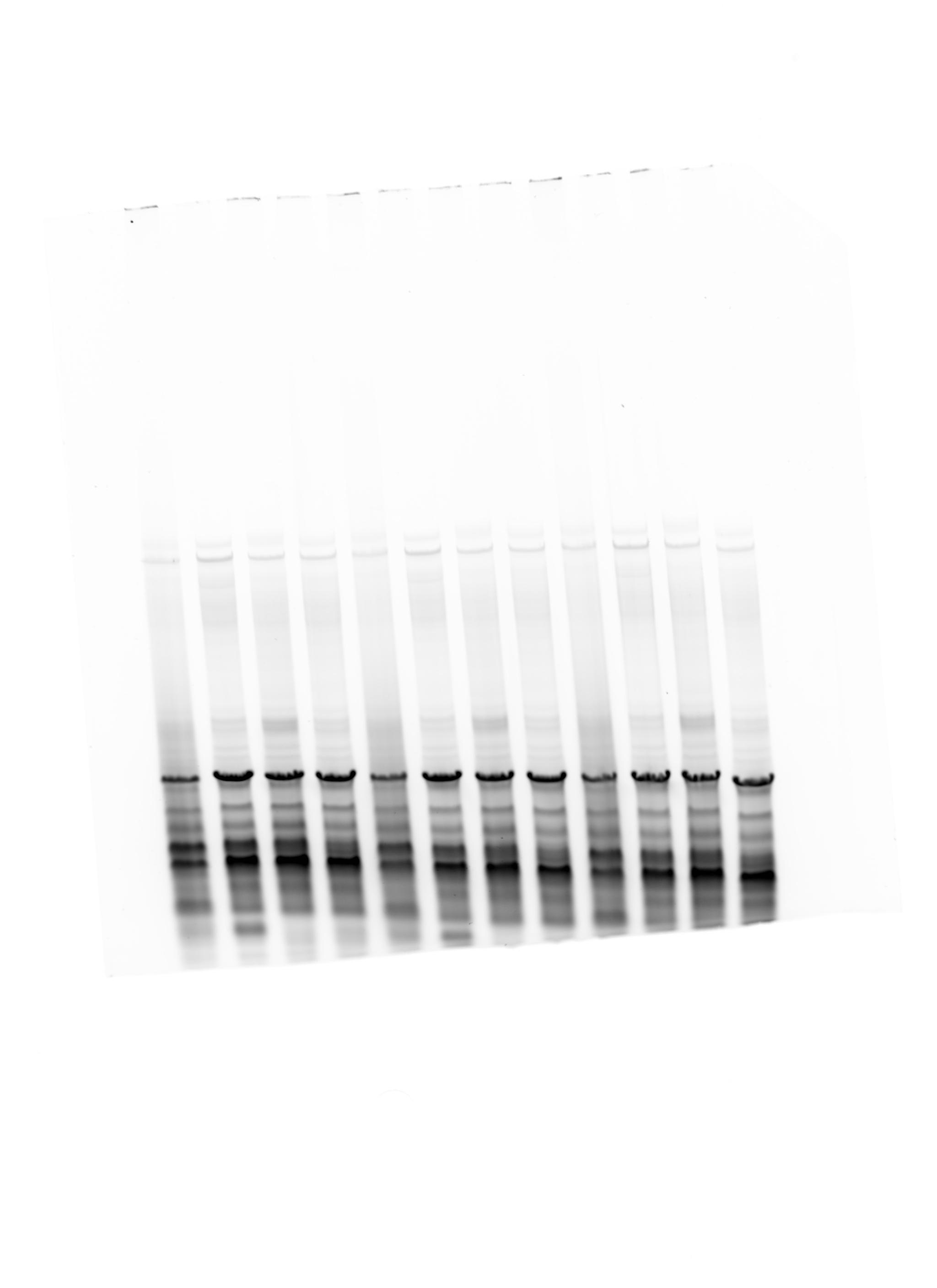

Supplement: Supplementary file 2 [file Image2.JPEG]

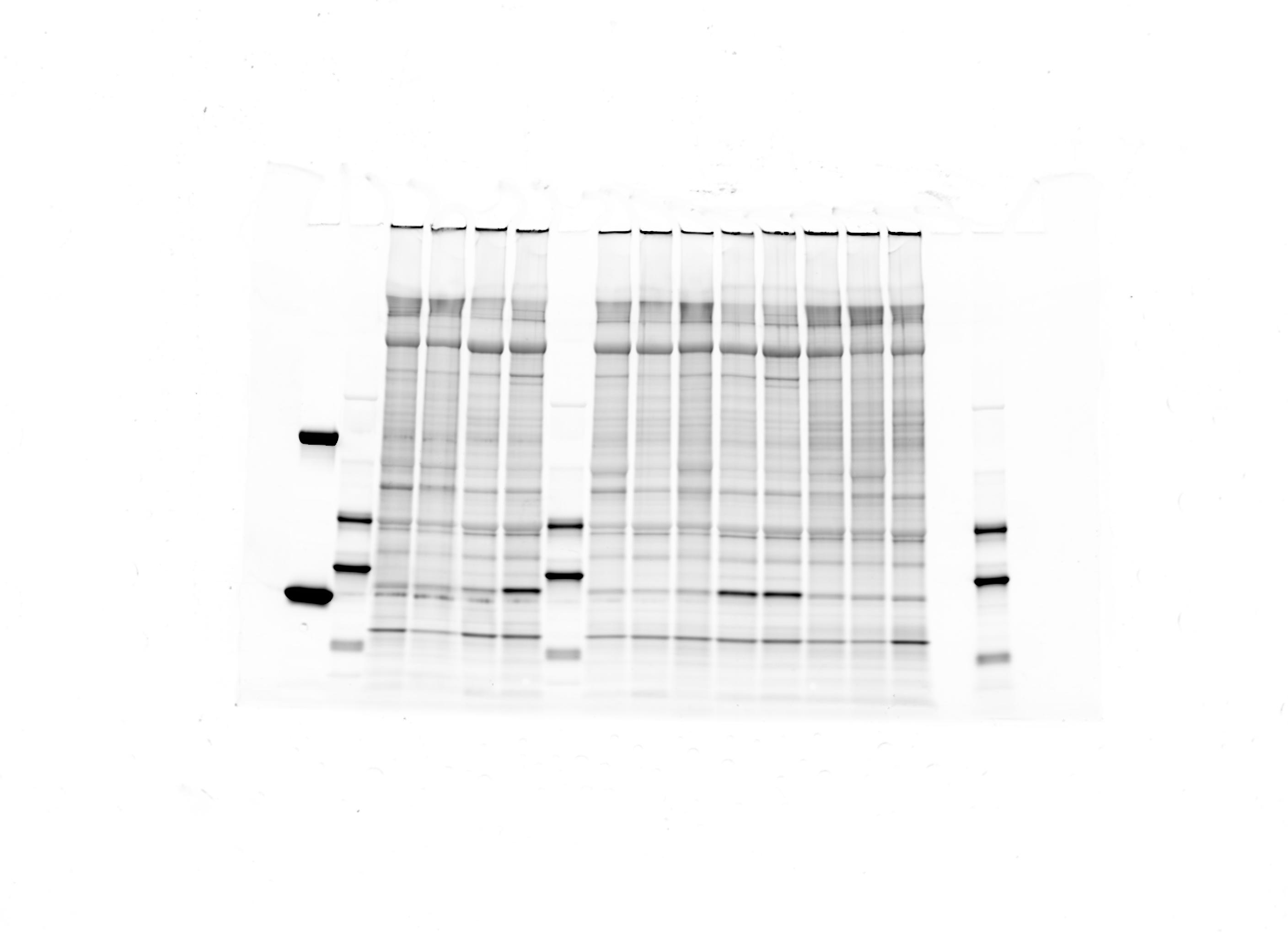

Supplement: Supplementary file 3 [file Image3.JPEG]

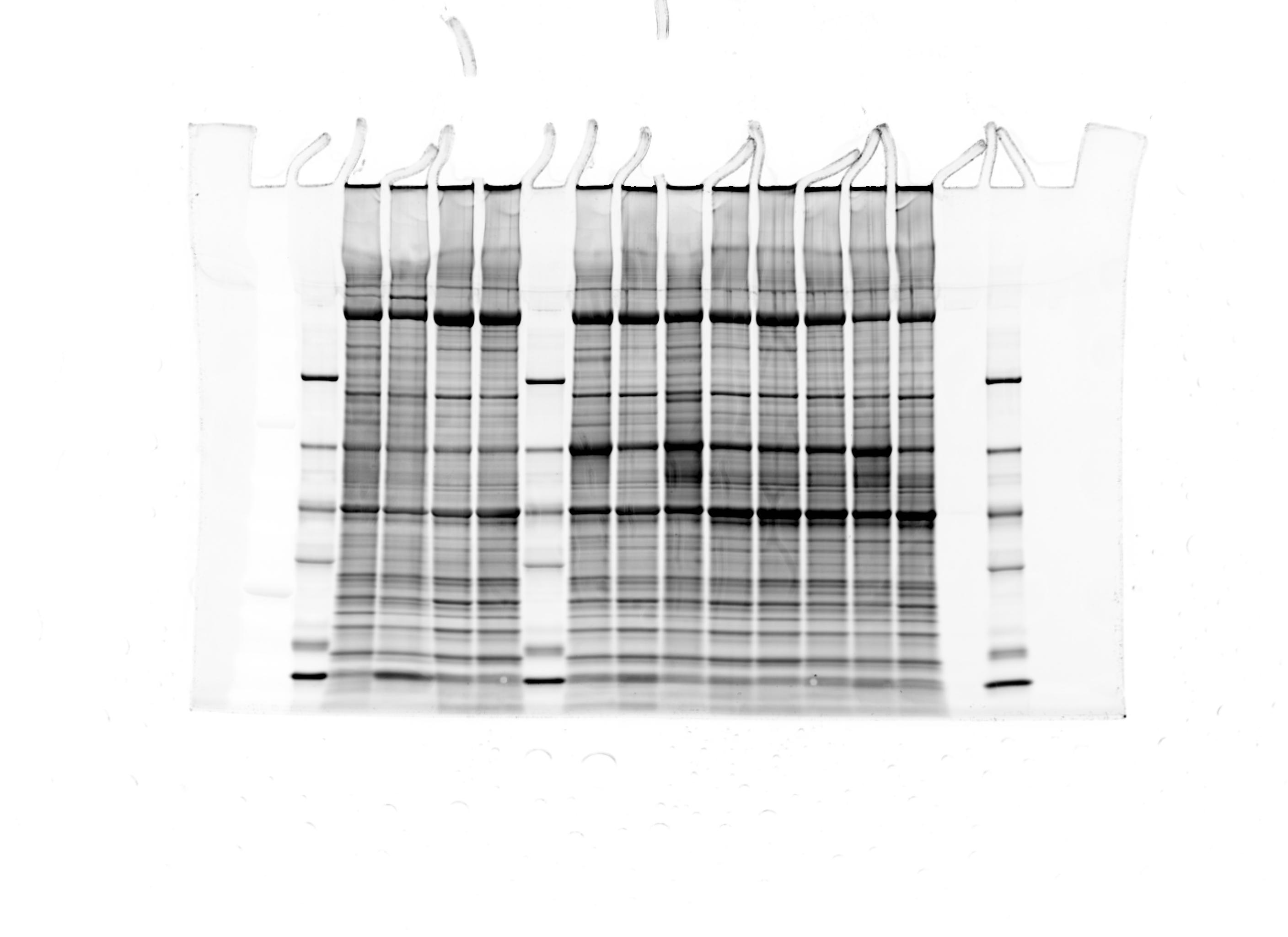

Supplement: Supplementary file 4 [file Image4.JPEG]

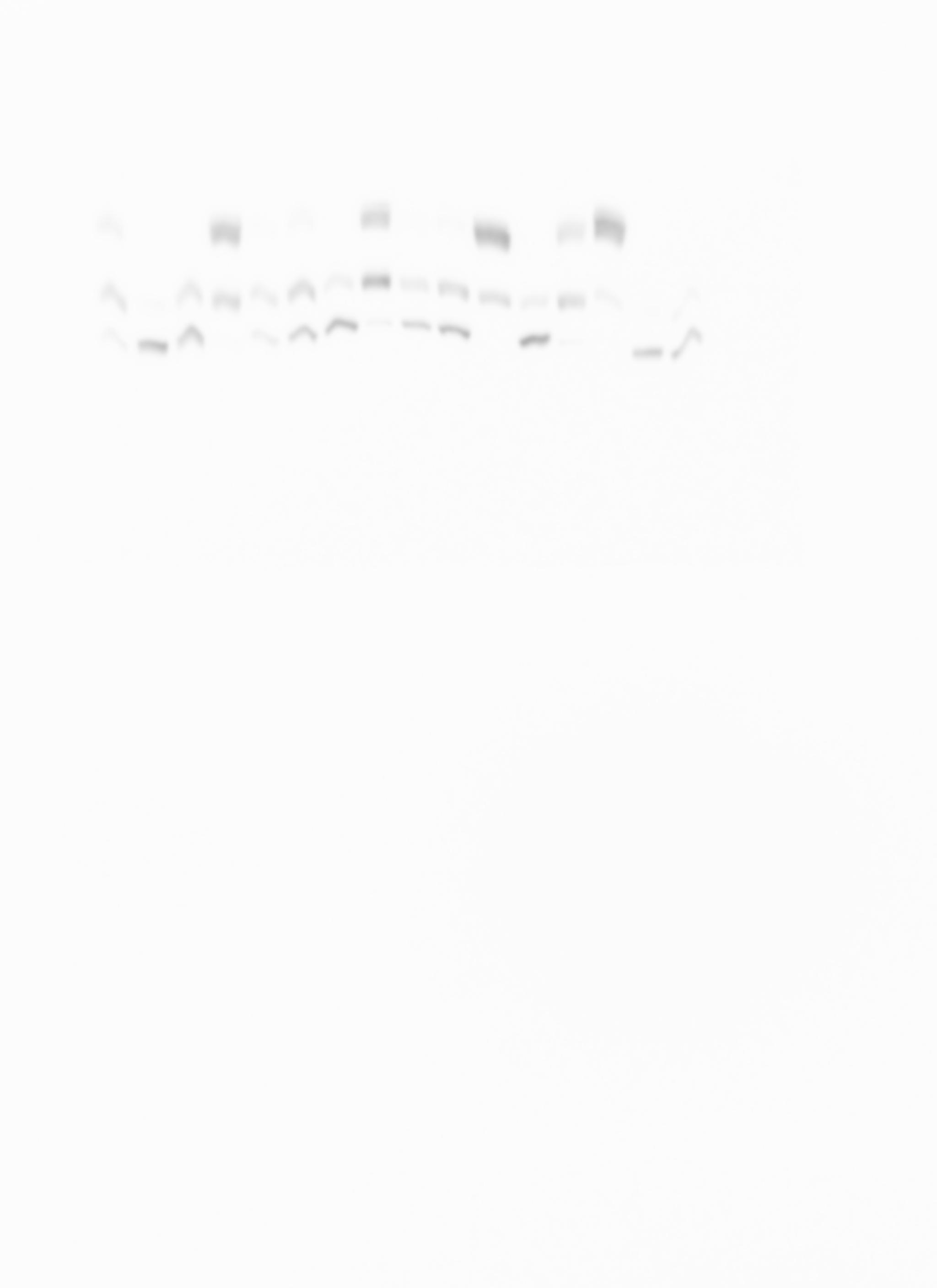

Supplement: Supplementary file 5 [file Image5.JPEG]

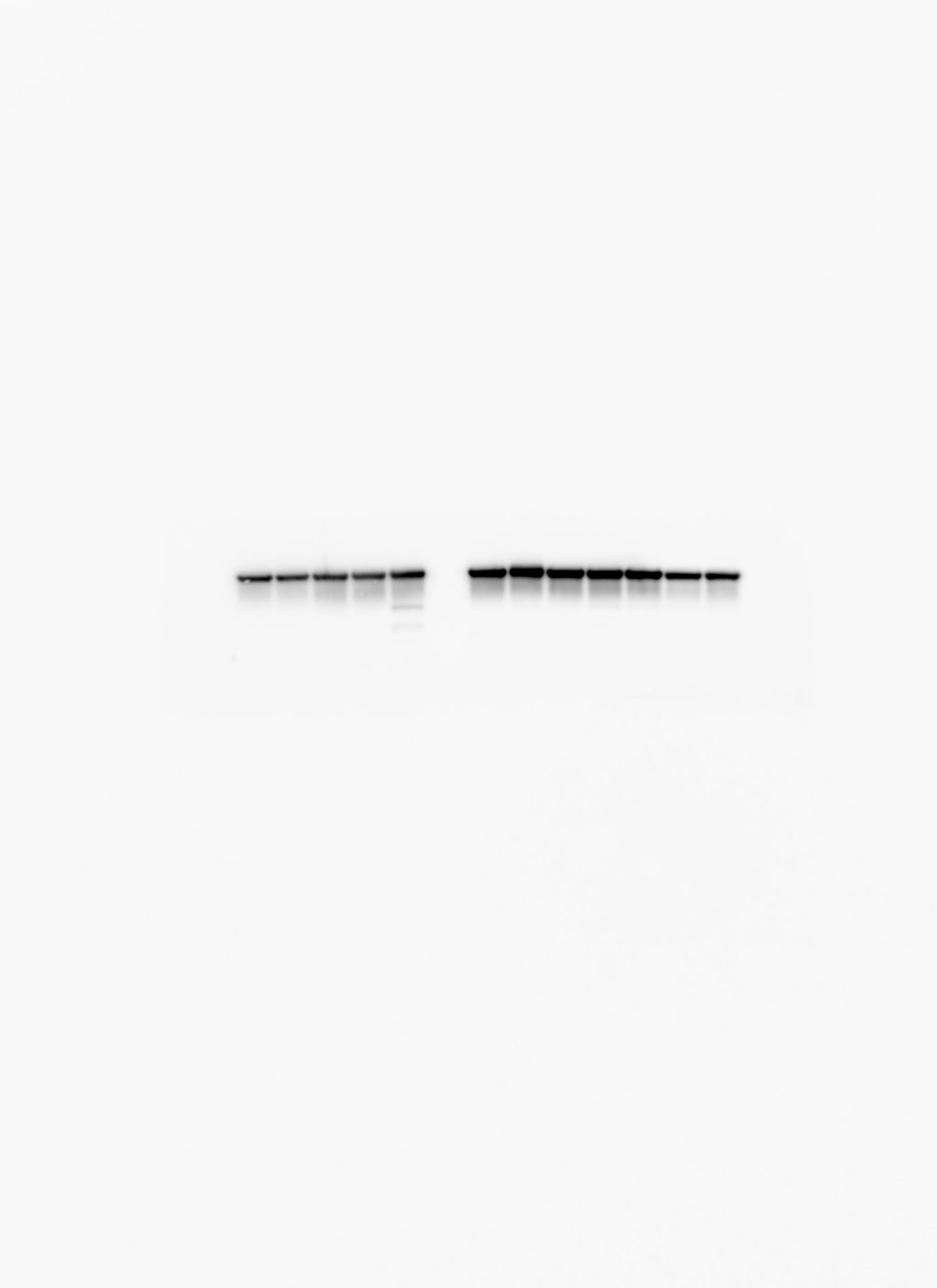

Supplement: Supplementary file 6 [file Image6.JPEG]

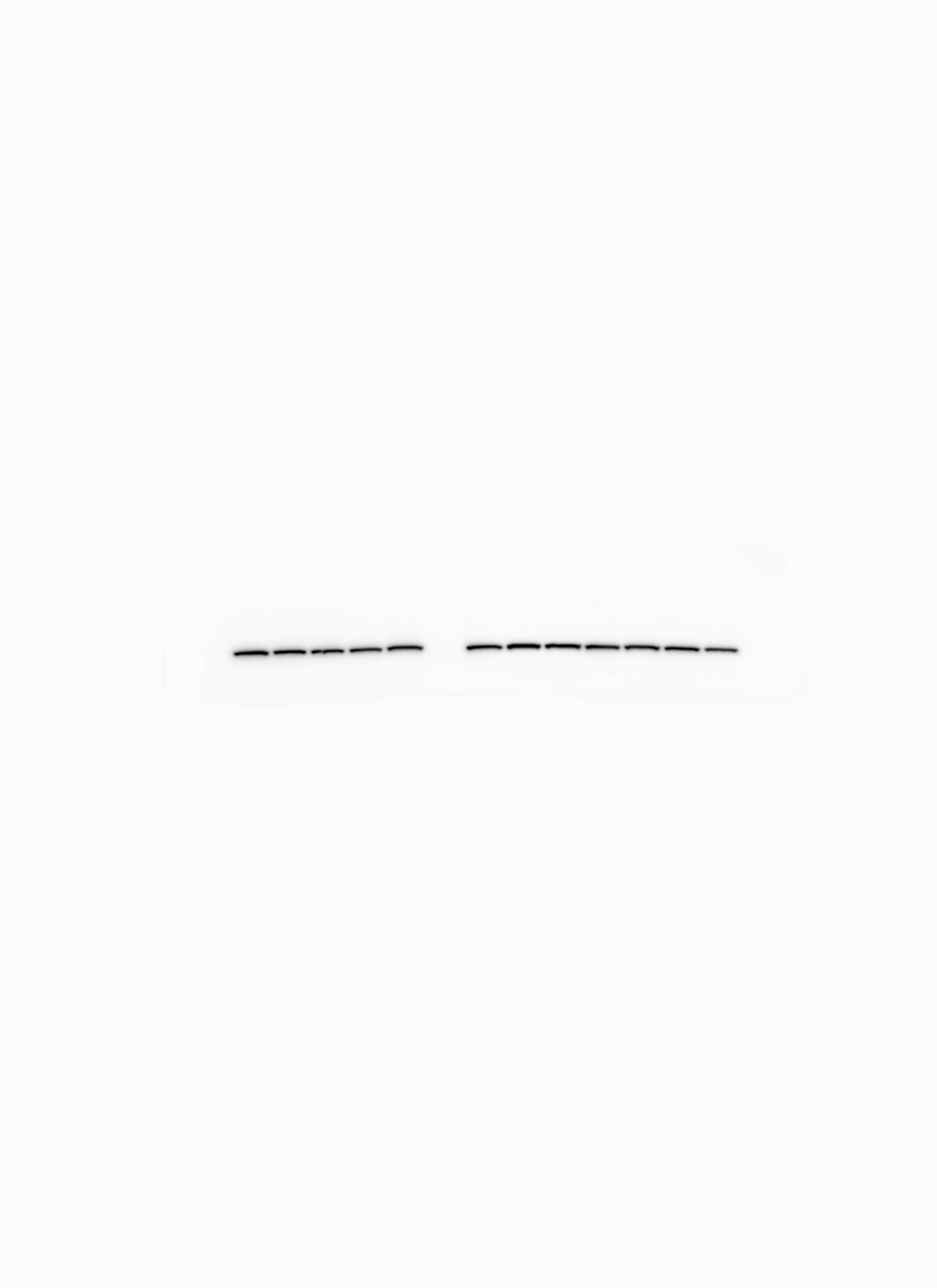

Supplement: Supplementary file 7 [file Image7.JPEG]

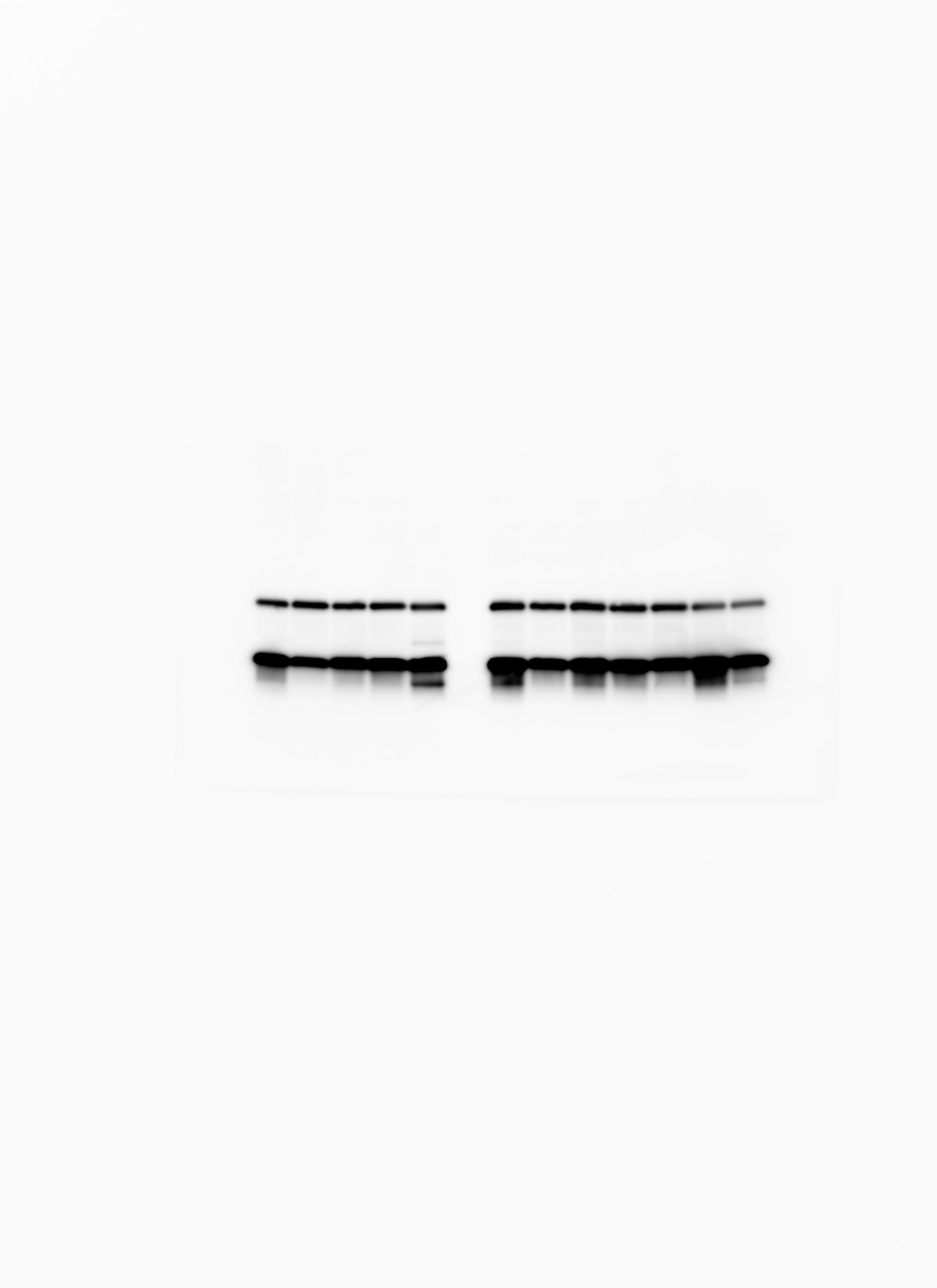

Supplement: Supplementary file 8 [file Image8.JPEG]

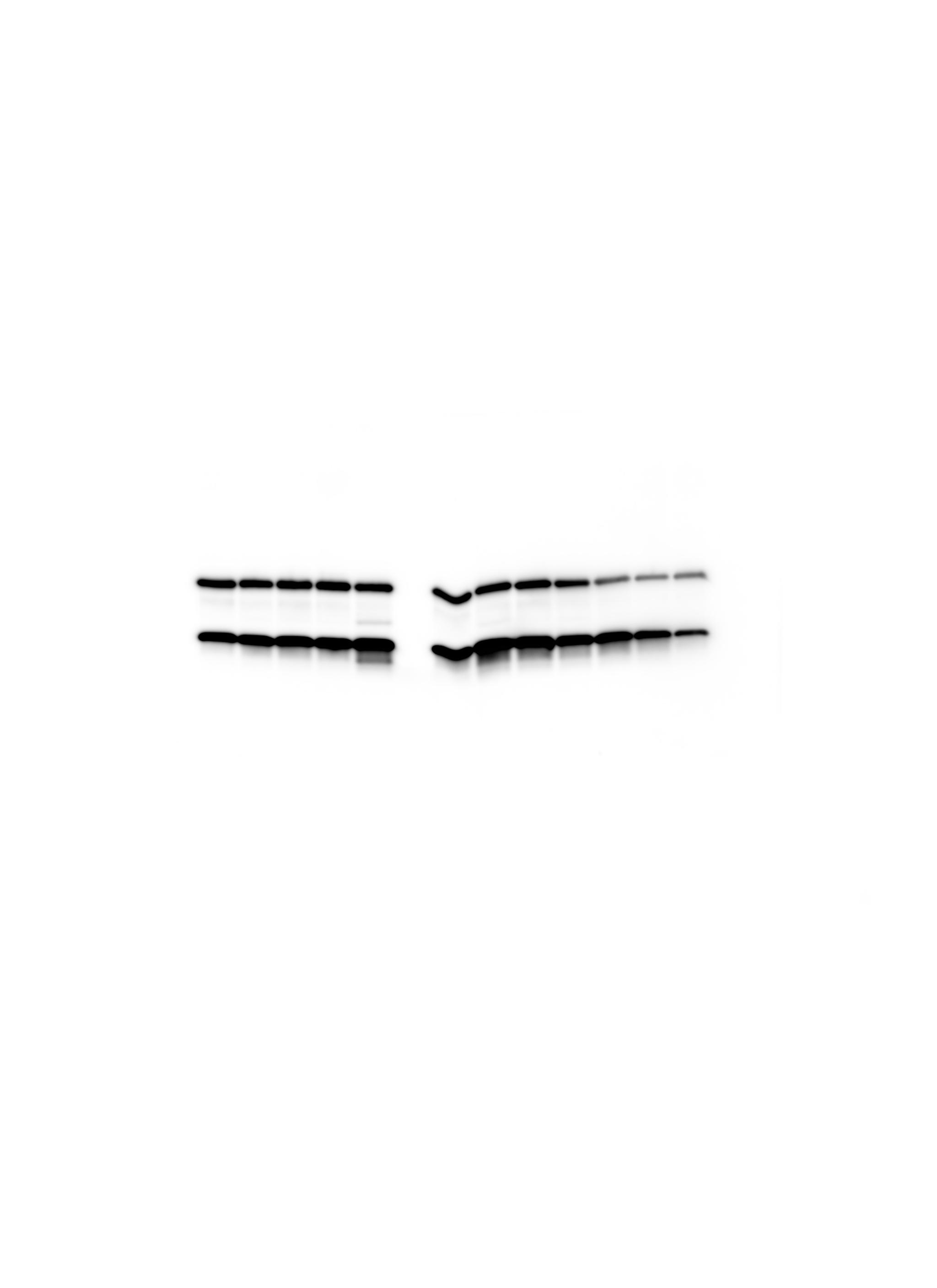

Supplement: Supplementary file 9 [file Image9.JPEG]

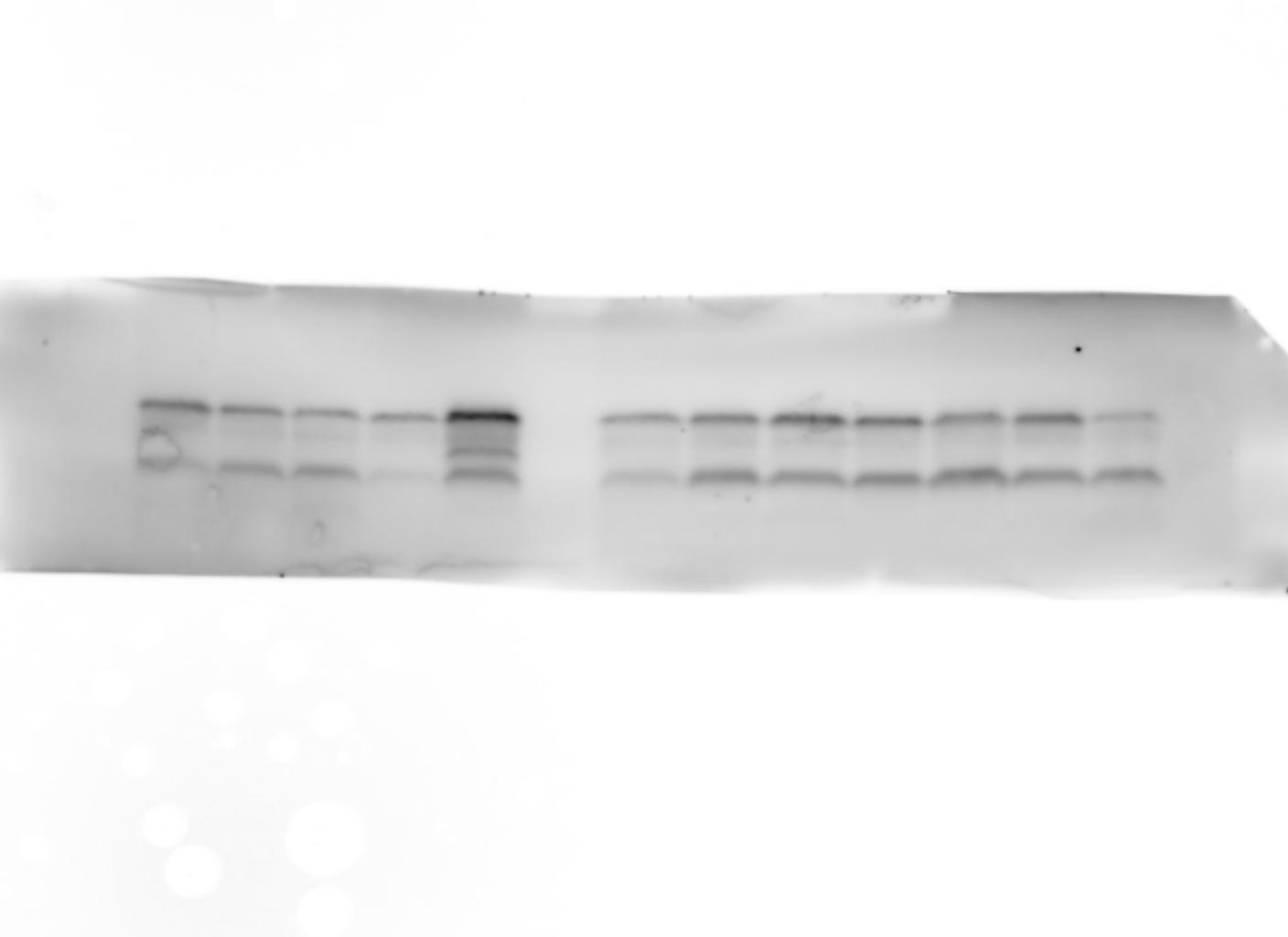

Supplement: Supplementary file 10 [file Image10.JPEG]

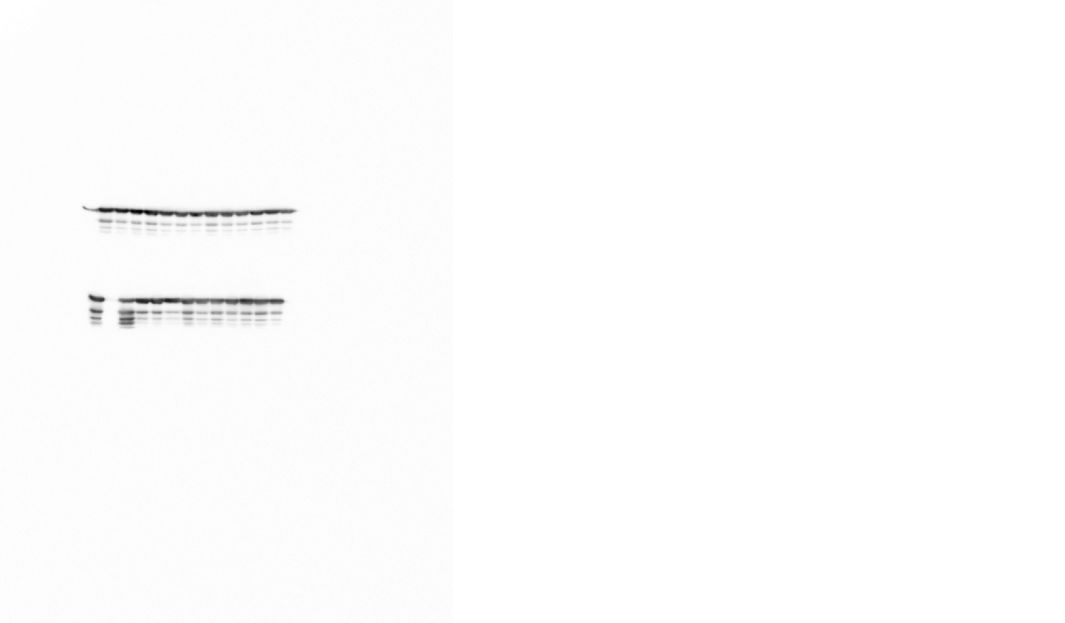

Supplement: Supplementary file 11 [file Image11.JPEG]

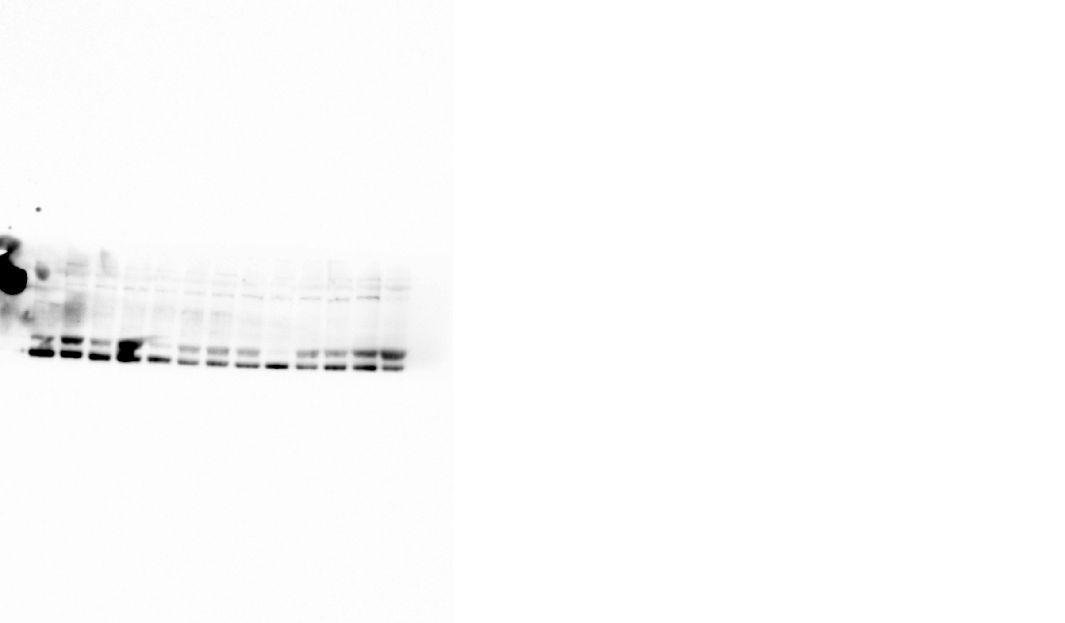

Supplement: Supplementary file 12 [file Image12.JPEG]
